# Numerical Computation of Homogeneous Slope Stability

**DOI:** 10.1155/2015/802835

**Published:** 2015-02-15

**Authors:** Shuangshuang Xiao, Kemin Li, Xiaohua Ding, Tong Liu

**Affiliations:** School of Mines, State Key Laboratory of Coal Resources and Safe Mining, China University of Mining and Technology, Xuzhou, Jiangsu 221116, China

## Abstract

To simplify the computational process of homogeneous slope stability, improve computational accuracy, and find multiple potential slip surfaces of a complex geometric slope, this study utilized the limit equilibrium method to derive expression equations of overall and partial factors of safety. This study transformed the solution of the minimum factor of safety (FOS) to solving of a constrained nonlinear programming problem and applied an exhaustive method (EM) and particle swarm optimization algorithm (PSO) to this problem. In simple slope examples, the computational results using an EM and PSO were close to those obtained using other methods. Compared to the EM, the PSO had a small computation error and a significantly shorter computation time. As a result, the PSO could precisely calculate the slope FOS with high efficiency. The example of the multistage slope analysis indicated that this slope had two potential slip surfaces. The factors of safety were 1.1182 and 1.1560, respectively. The differences between these and the minimum FOS (1.0759) were small, but the positions of the slip surfaces were completely different than the critical slip surface (CSS).

## 1. Introduction

Slide is one of the most common types of natural hazards on slopes, which might lead to considerable casualties and economic loss [1, 2]. To guarantee the stability of a homogenous slope, the circular arc method is traditionally used for stability analysis. The two main objectives of slope stability analysis are calculating FOS for a given slip surface and determining the CSS for a given slope [3]. The Swedish circle method, the so-called Fellenius slice method, is a widely used classic algorithm for calculating the FOS of an arc sliding slope [4]. To avoid repeated slices for summation, Zhang et al. derived the integral expression equations for the FOS using the Swedish circle method and proposed different solutions for minimum factors of safety and for critical slip surfaces [5–8]. However, all of these efforts were suitable for an integral over a slope with a plane surface [9]. For a slope with a complex surface, due to variations of the slope geometry, the integral results might be different, in which case, the integral expression equations derived by Zhang et al. could become problematic.

Furthermore, complex slope geometry could result in multiple slip surfaces, which have a relatively small difference between their FOS and the minimum FOS. However, the positions of the slip surfaces are completely different from that of the CSS. If only the CSS was identified and supported, the slope could slide along other surfaces [10, 11]. Therefore, all potential slip surfaces should be identified and supported or partially supported according to the actual situation to ensure the stability of the slope. Moreover, when the slope geometry is complex, there may be an intercept between the precalculated slip surface and the slope, that is, a broken arc [12, 13], which could further complicate the stability computation.

The solution methods for an integral expression equation of the slope FOS include analytical methods [9], exhaustive method [14], numerical optimization method [15], random search method [16], and artificial intelligent search method [17]. The analytical method uses a partial derivative of the expression equation through finding the extreme values of the function. This method requires multiple cross iterations for analyzing a pure clay slope [18], and is even less effective in analyzing slopes with complex surface, while other methods usually require a search of the variables to perform a precalculation. In this regard, there are currently three typical selection methods for variable searching: (1) the horizontal coordinate *x*_*c*_, vertical coordinate *y*_*c*_ of the center of critical circular arc, and the radius *R* [19, 20]; (2) the horizontal coordinates of the intercepts of the slip surface with a slope top and bottom surfaces, that is, *x*_*u*_ and *x*_*b*_, and the vertical coordinate of the center of critical circular arc, *y*_*c*_ [21]; (3) the horizontal coordinates of the intercepts of the slip surface with the slope top and bottom surfaces and the arc height *h*_*a*_ [22] or the horizontal coordinate of the intercept between the *x*-axis and the tangent that is at the intercept between the slip surface and the slope top, *x*_*t*_.

In the first method, the range of *x*_*c*_, *y*_*c*_, and *R* is determined empirically. Unreasonable ranges can lead to a missed optimum solution. In the second method, determination of the range of the three parameters *x*_*u*_, *x*_*b*_, and *y*_*c*_ is also needed. But the location of the intercept between the slip surface and slope top and bottom is easily controlled and the range of *x*_*u*_ and *x*_*b*_ is relatively easy to determine. In the third method, the range of *x*_*u*_ and *x*_*b*_ needs to be estimated and the range of *h*_*a*_ or *x*_*t*_ is known. Although the range of *h*_*a*_ or *x*_*t*_ does not need to be estimated, the variation of *h*_*a*_ or *x*_*t*_ corresponds to the variation of *R*. For instance, the varying range of *h*_*a*_ is from *h*_*a*max⁡_ to zero, which corresponds to the range if *R* would be from *R*_min⁡_ to an infinitely large value, which is essentially equivalent to selecting *R* as the searching variable and letting its range be [*R*_min⁡_, +*∞*]. Therefore, this approach does not improve the performance of the first method but increases the search range considerably, which adds unnecessary searching. Although there is no essential difference among these three methods, the second method selects relatively direct searching variables and the range is fairly easy to control.

This paper utilized the concept of these described integral methods and derived the integral expression equation of the FOS for a homogenous slope with complex slope geometry. This study used the second method to select searching variables and applied an EM and PSO to solve for the minimum FOS. Using the method presented in this paper, the minimum FOS of a homogenous slope can be effectively calculated. The CSS, as well as other potential slip surfaces, can be identified, which provides a scientific basis for slope support.

## 2. Derivation of the Integral Expression Equation for Slope FOS

### 2.1. Integral Expression Equation for the Overall Slope FOS

**Figure 1:**
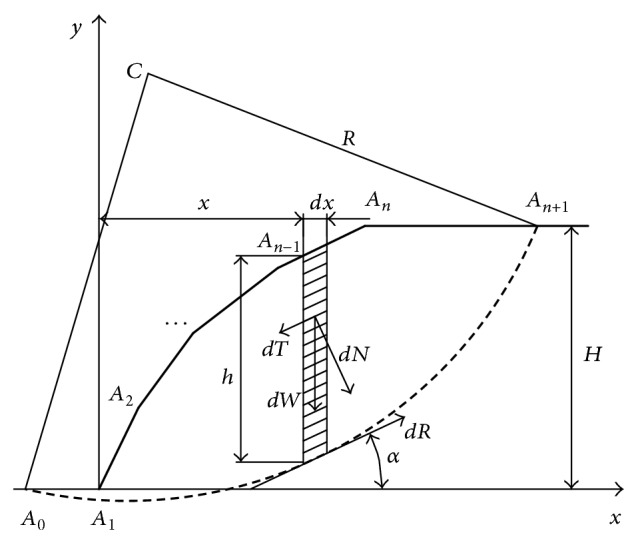
Coordinate system for the slopes stability analysis and forces acting on differential slices.

For a slope with complex geometry, the line of the slope surface can be assumed to consist of several component lines [23]. For the slope shown in Figure 1, the slope surface is composed of *n* − 1 lines *A*_1_ *A*_2_, *A*_2_ *A*_3_,…, *A*_*n*−1_ *A*_*n*_. The slope is assumed to be a homogenous slope and the surfaces of the slope's top and bottom are planes. Moreover, it is also assumed that there is no effect of slope loading or groundwater influence. The height of slope is *H*, the unit weight of the slope soil is *γ*, the internal friction angle is *φ*, and the cohesion is *c*. The coordinate system is established with the origin selected at the toe of slope *A*_1_, shown in Figure 1. Let the radius of the critical arc be *R* and the center of the arc *C*(*x*_*c*_, *y*_*c*_). The intercepts between the slip surface and the surfaces of slope's top and bottom are *A*_*n*+1_ and *A*_0_, respectively. The coordinate of *A*_*i*_ is (*x*_*i*_, *y*_*i*_)  (*i* = 0,1, 2,…, *n* + 1); thus *y*_0_ = 0, *x*_1_ = 0, *y*_1_ = 0, *y*_*n*_ = *H*, and *y*_*n*+1_ = *H*.

Assuming the equation for line *A*_*i*_ *A*_*i*+1_,

(1) $$\begin{array}{c} y_{xi}=k_{i}x+b_{i},\;x_{i}\leq x\leq x_{i+1}, \end{array}$$

where

(2) ki=yi+1−yixi+1−xi,  bi=yixi+1−yi+1xixi+1−xiiiiiiiiiiiiiixi≠xi+1, i=0,1,2,…,n.

The equation for arc *A*_0_ *A*_*n*+1_ was

(3) $$\begin{array}{c} y_{a}=y_{c}-\sqrt{R^{2}-{\left(x-x_{c}\right)}^{2}},\;x_{0}\leq x\leq x_{n+1}. \end{array}$$

For the infinitely small slide bar, the width was assumed to be *dx* and the height was *h*; thus

(4) $$\begin{array}{c} h=y_{xi}-y_{a},\;x_{i}\leq x\leq x_{i+1}. \end{array}$$

The weight of slide bar *dW* = *γhdx*. Antislide force and downslide force were

(5) $$\begin{array}{c} dR=c\text{sec}\;\alpha dx+\gamma h\tan\varphi \cos\alpha dx\\ dT=\gamma h\sin\alpha dx, \end{array}$$

where sin*α* = (*x* − *x*_*c*_)/*R*.

The FOS *F* of the slope is the ratio of the torque at the center of arc *C* between all antislide forces and downslide forces; thus the overall FOS of the slope was

(6) $$\begin{array}{c} F_{z}=\frac{M_{rz}}{M_{sz}}=\frac{cI_{rz}+\gamma \tan\varphi I_{cz}}{\gamma I_{sz}}. \end{array}$$

According to (1)–(5), the integration yielded

(7) Irz=∫x0xn+1Rsec αdx=R2δn+1−δ0Icz=∑i=0n∫xixi+1Ryxi−yacos⁡αdx=Pn+1−P0+∑i=0n13kiSi3−Si+13        +12kixc+bi−ycTi+1−TiIsz=∑i=0n∫xixi+1Ryxi−yasinαdx=13S03−13Sn+13+∑i=0nWi,

where

(8) δi=arcsinxi−xcR,  Si=R2−xi−xc2,Ti=(xi−xc)Si+R2δi,  Pi=R2xi−13xi−xc3,Wi=13kixi+13−xi3+12bi−kixc−ycxi+12−xi2 +xcyc−bixi+1−xi    (i=0,1,2,…,n).

When *x*_*i*_ = *x*_*i*+1_, the line equation was *x* = *x*_*i*_, *y*_*i*_ ≤ *y* ≤ *y*_*i*+1_; the corresponding integral in the range of this line would be zero.

### 2.2. Integral Expression Equation for the Partial Slope FOS

**Figure 2:**
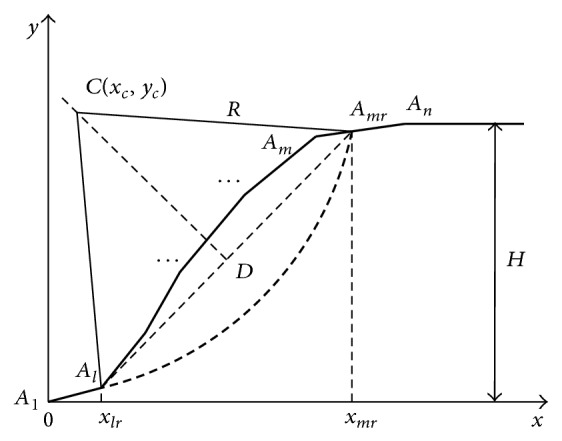
Sketch of slopes partial stability analysis.

Figure 2 shows that the slip surface intercepts with the slope surface. Assuming that top and bottom intercepts have coordinates of (*x*_*mr*_, *y*_*mr*_) and (*x*_*lr*_, *y*_*lr*_), thus *x*_*l*_ ≤ *x*_*lr*_ ≤ *x*_*l*+1_  (0 ≤ *l* ≤ *n* − 2), *x*_*m*_ ≤ *x*_*mr*_ ≤ *x*_*m*+1_  (*l* + 2 ≤ *m* ≤ *n*; in addition, *m* < *n* when *l* = 0).

According to (1)–(5), the partial FOS can be obtained through integration:

(9) $$\begin{array}{c} F_{j}=\frac{M_{rj}}{M_{sj}}=\frac{cI_{rj}+\gamma \tan\varphi I_{cj}}{\gamma I_{sj}}, \end{array}$$

where

(10) Irj=∫xlxm+1Rsec αdx=R2(δm+1−δl)Icj=∑i=lm∫xixi+1Ryxi−yacos⁡αdx=Pm+1−Pl+∑i=lm13kiSi3−Si+13        +12kixc+bi−ycTi+1−TiIsj=∑i=lm∫xixi+1Ryxi−yasinαdx=13Sl3−13Sm+13+∑i=lmWi,

where *x*_*m*+1_ = *x*_*mr*_ in *δ*_*m*+1_, *S*_*m*+1_, *T*_*m*+1_, *P*_*m*+1_, and *W*_*m*_ and *x*_*l*_ = *x*_*lr*_ in *δ*_*l*_, *S*_*l*_, *T*_*l*_, *P*_*l*_, and *W*_*l*_.

## 3. Calculation of Slope FOS

### 3.1. Calculation of Overall Slope FOS

Equations (7) were substituted into (6). Equations (10) were substituted into (16). For the known slope, its FOS *F* is the function of the horizontal and vertical coordinates of the center of the critical arc, *x*_*c*_ and *y*_*c*_, as well as the radius *R*. However, it is difficult to obtain the minimum value of this function using the analytical method. The selection of the searching variable for the precalculation is needed to solve the problem.

From the geometrical relationship in Figure 1, it is known that

(11) $$\begin{array}{c} x_{c}=\frac{H^{2}+{x_{n+1}}^{2}-{x_{0}}^{2}}{2\left(x_{n+1}-x_{0}\right)}-\frac{Hy_{c}}{x_{n+1}-x_{0}}, \end{array}$$

(12) $$\begin{array}{c} R=\sqrt{{\left(x_{n+1}-x_{c}\right)}^{2}+{\left(y_{c}-H\right)}^{2}}. \end{array}$$

For a known slope, when *x*_0_, *x*_*n*+1_, and *y*_*c*_ are determined, *x*_*c*_ and *R* can be determined using (11) and (12), respectively; that is, the CSS can be obtained. Therefore, *x*_0_, *x*_*n*+1_, and *y*_*c*_ can be selected as the searching variables, in which *x*_*n*+1_ ≥ *x*_*n*_, *x*_0_ ≤ 0. To reduce the searching range, a restrained range of value of *x*_0_, *x*_*n*+1_, *y*_*c*_ should be empirically evaluated to ensure that *y*_*c*_ ≤ *y*_*c*max⁡_, *x*_0_ ≥ *x*_0min⁡_, *x*_*n*+1_ ≥ *x*_(*n*+1)max⁡_. In addition, to avoid occurrence of a broken arc, $\sqrt{{\left(x_{i}-x_{c}\right)}^{2}+{\left(y_{i}-y_{c}\right)}^{2}}\leq R$  (*i* = 1,2,…, *n*) should be satisfied. Under this circumstance, solving for the minimum FOS requires solving the following constrained nonlinear programming problem:

(13) Fzm=min⁡Fz(x0,xn+1,yc)s.t. x0min⁡≤x0≤0  xn≤xn+1≤xn+1max⁡  yc≤ycmax⁡  xi−xc2+yi−yc2≤R  i=1,2,…,n.

When the determined critical circular arc center is on the boundary of the search region, such as *y*_*c*_ = *y*_*c*max⁡_, *x*_0_ = *x*_0min⁡_, or *x*_*n*+1_ = *x*_(*n*+1)max⁡_, the searching region should be expanded accordingly. Otherwise, this could result in missing the optimum solution due to the small range of the value. Taking *y*_*c*max⁡_ as an example, when the vertical coordinate of the critical arc center *y*_*c*_ = *y*_*c*max⁡_, (14) can be used for adjustment. Consider

(14) $$\begin{array}{c} y_{c\max}=\left(1+\lambda \right)y_{c\max}, \end{array}$$

where *λ* is a coefficient.

This study applied an EM and PSO to solve (13). The result yielded from the EM was compared in order to validate the result of the PSO.

#### 3.1.1. Exhaustive Method

**Figure 3:**
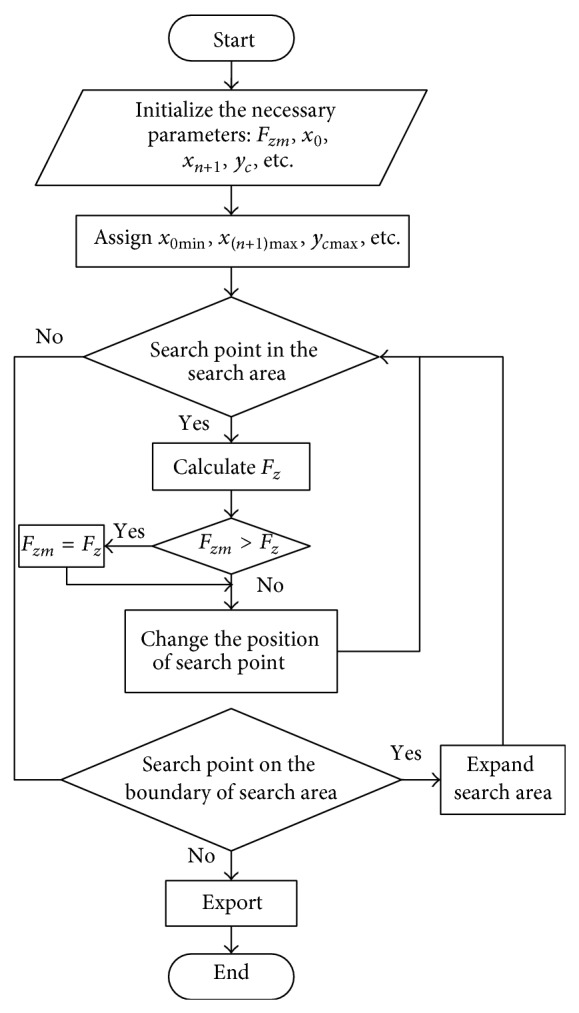
Flow chart of calculating the minimum FOS.

The EM is a method that allows the range of *x*_0_, *x*_*n*+1_, and *y*_*c*_ to vary according to a specified step interval. The FOS *F*_*z*_ should be solved at every step for each value, in which the minimum value would be the minimum FOS *F*_*zm*_. The detailed solving procedure is shown in Figure 3. This procedure can be easily carried out using a set of programmed computer codes.

#### 3.1.2. Particle Swarm Optimization Algorithm

The PSO is a swarm intelligent heuristic algorithm that was proposed by Dr. Eberhart and Dr. Kennedy [24]. It utilizes individual coordination and information sharing in the swarm to seek the optimum solution through iteration. The concept of PSO is simple. There are few adjusting parameters and the convergence speed is fast, so it has been widely used in discrete and continuous optimization problems [25, 26].

During the searching process of the CSS on the slope using the PSO, each precalculated slip surface can be treated as a particle. The number of searching variables is the dimension of the searching space. The FOS function *F*_*z*_ is the fitness function. Assuming that the searching space is *D*-dimension, the particle swarm consists of *N* particles. The location of the *i*th particle at the *t*th time step is **O**_*i*_^*t*^ = (*O*_*i*1_^*t*^, *O*_*i*2_^*t*^,…, *O*_*iD*_^*t*^); the velocity is **V**_*i*_^*t*^ = (*V*_*i*1_^*t*^, *V*_*i*2_^*t*^,…, *V*_*iD*_^*t*^). Thus the procedure of searching for the CSS of the slope using PSO can be expressed as follows.

(1) Parameter initialization, including the swarm size *N*, particle location **O**_*i*_^*t*^ and velocity **V**_*i*_^*t*^, the maximum velocity of the particle **V**_max⁡_, and maximum iteration step *t*_max⁡_.

(2) Examination of the particle location to check if the geometric condition is satisfied, that is, to ensure a broken arc does not occur in the determined slip surface: if the conditions were satisfied, the calculation would enter step (3); otherwise, the particle location should be adjusted to satisfy the geometric condition and then enter step (3).

(3) Calculating the fitness function value of each particles, that is, calculating the FOS of each slip surface *F*_*zi*_^*t*^ = *F*_*z*_(*O*_*i*1_^*t*^, *O*_*i*2_^*t*^,…, *O*_*iD*_^*t*^).

(4) Comparing the fitness function value of each particle *F*_*zi*_^*t*^ with the corresponding fitness function value at the best position where the particle passed *F*_*zpi*_: if *F*_*zi*_^*t*^ ≤ *F*_*zpi*_, the particle passed best position of **O**_*pi*_ = **O**_*i*_^*t*^ and the optimal fitness function value of the particle *F*_*zpi*_ = *F*_*zi*_^*t*^; otherwise, **O**_*pi*_ and *F*_*zpi*_ maintained the same values.

(5) Comparing the fitness function value of each particle *F*_*zi*_^*t*^ with the fitness function value at the overall best position: if *F*_*zi*_^*t*^ ≤ *F*_*zg*_, all particles passed the best position at **O**_*g*_ = **O**_*i*_^*t*^ and the overall fitness function value was *F*_*zg*_ = *F*_*zi*_^*t*^; otherwise, **O**_*g*_ and *F*_*zg*_ maintained the same values.

(6) According to (15) and (16), the velocity and location of each particle were updated:

(15) Vit+1=wVit+c1r1Opi−Oit+c2r2Og−Oit,Vijt+1=Vijt+1if  Vijt+1  ≤Vjmax⁡Vijt+1Vijt+1Vjmax⁡if  Vijt+1  >Vjmax⁡,

(16) $$\begin{array}{c} O_{i}^{t+1}=O_{i}^{t}+V_{i}^{t+1}, \end{array}$$

where *i* = 1,2,…, *N*,  *j* = 1,2,…, *D*; *c*_1_ and *c*_2_ are the acceleration coefficients, with a typical value of *c*_1_ = *c*_2_ = 2. *r*_1_ and *r*_2_ are random number in the range of [0,1]. *w* is the inertial factor, which is determined using (17) [27].

In (16), when the particle runs out of the searching range, that is, *O*_*ij*_^*t*+1^ > *O*_*j*max⁡_ or *O*_*ij*_^*t*+1^ < *O*_*j*min⁡_, the “reflecting walls” approach would be used [28]. Let *V*_*ij*_^*t*^ = −*V*_*ij*_^*t*^ and *O*_*ij*_^*t*+1^ = *O*_*ij*_^*t*^ − *V*_*ij*_^*t*^. Consider

(17) $$\begin{array}{c} w=w_{\max}-t\times \frac{w_{\max}-w_{\min}}{t_{\max}}, \end{array}$$

where *w*_max⁡_ is initial iteration inertial factor and *w*_min⁡_ is the inertial factor at the final iteration. Let *w*_max⁡_ = 0.9, *w*_min⁡_ = 0.4 [29]; *t*_max⁡_ is the maximum iteration step and *t* is the current interaction step.

(7) If *t* < *t*_1_, let *t* = *t* + 1. Then the procedure steps back to (2); otherwise, it enters the next step.

(8) If the overall optimal fitness function value satisfies (18) [30, 31] or the iteration *t* > *t*_max⁡_, the searching process would be ended. At this point, *F*_*zg*_ is the minimum FOS and the corresponding slip surface is the CSS. Otherwise, let *t* = *t* + 1, *t*_1_ = *t*_1_ + *t*_*c*_, *F*_*zgo*_ = *F*_*zg*_, and the procedure enter step (2) and the next iteration. Consider

(18) $$\begin{array}{c} \left|F_{zgo}-F_{zg}\right|\leq \varepsilon , \end{array}$$

where *F*_*zgo*_ and *F*_*zg*_ are the overall optimal fitness function values at the iteration steps *t*_1_ and *t*_1_ + *t*_*c*_, respectively. *ε* is the expected minimum degree of error. In (18), after *t*_*c*_ iteration steps, the variation of the overall optimal fitness function value becomes small.

**Figure 4:**
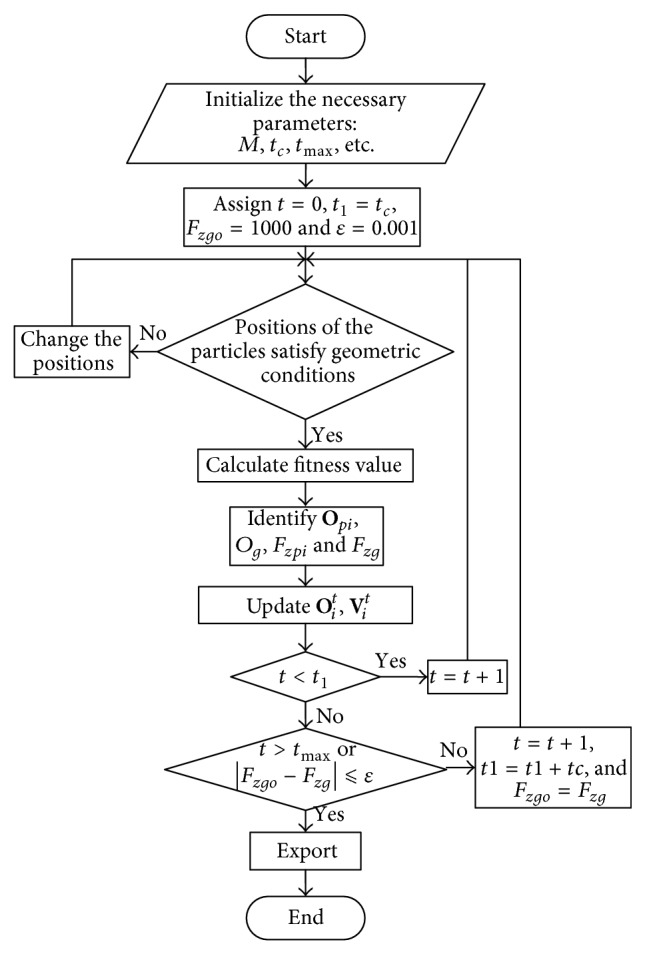
Flow chart of the particle swarm optimization.

The detailed procedure is shown in Figure 4.

Given a swarm size *N*, maximum iteration step *t*_max⁡_ can be determined through sensitivity analysis [32].

### 3.2. Calculation of the Partial Slope FOS

It can be seen from the geometric relationship in Figure 2 that

(19) $$\begin{array}{c} x_{c}=\frac{{y_{mr}}^{2}-{y_{lr}}^{2}+{x_{mr}}^{2}-{x_{lr}}^{2}}{2\left(x_{mr}-x_{lr}\right)}-\frac{y_{mr}-y_{lr}}{x_{mr}-x_{lr}}y_{c}, \end{array}$$

(20) $$\begin{array}{c} R=\sqrt{{\left(x_{mr}-x_{c}\right)}^{2}+{\left(y_{c}-x_{mr}\right)}^{2}}. \end{array}$$

For a known slope, when *x*_*lr*_ and *x*_*mr*_ are known, *y*_*lr*_ and *y*_*mr*_ can also be determined. If *y*_*c*_ were also known, *x*_*c*_ and *R* could be determined through (19) and (20), respectively. *x*_*lr*_, *x*_*mr*_, and *y*_*c*_ can be selected as the searching variables. Since the range of *x*_*mr*_ and *x*_*lr*_ is known, that is, *x*_*m*_ ≤ *x*_*mr*_ ≤ *x*_*m*+1_ and *x*_*l*_ ≤ *x*_*lr*_ ≤ *x*_*l*+1_, therefore, selecting the value range for *y*_*c*_ to let *y*_*c*_ ≤ *y*_*c*max⁡_ is the only requirement. Under this circumstance, solving for the minimum FOS requires solving the following constrained nonlinear programming problem:

(21) Fjm=min⁡Fjxmr,xlr,ycs.t. xl≤xlr≤xl+1  xm≤xmr≤xm+1  yc≤ycmax⁡  xi−xc2+yi−yc2≤R,

where 0 ≤ *l* ≤ *n* − 2, *l* + 2 ≤ *m* ≤ *n*, *m* − *l* < *n*, and *i* = *l* + 1,  *l* + 2,…, *m*.

The solving method for (21) is the same with that of (13), which is not repeated here. For the slope with *n* points on the slope surface shown in Figure 2, there are totally *n*(*n* − 1)/2 − 1 possible partial slip surfaces; that is, there are totally *n*(*n* − 1)/2 − 1 possible values for *m* and *l*. Equation (21) can be used to calculate the minimum FOS for each situation.

## 4. Case Study

### 4.1. Simple Slope Calculation

To validate the calculation method for the slope FOS in this study, a set of computational programs was developed. The EM and PSO were used to calculate the FOS for 5 simple slopes found in the literature. The results were compared with those from the literature and were listed in Table 1. It should be noted that the FOS of the slope was calculated using the variable metric method (VMM) [14], analytical method 1 (AM1) [7], analytical method 2 (AM2) [6], ordinary method (OM) [33], and genetic algorithms (GA) [34] reported in the literature.

In Table 1, it can be seen that the computational results from the EM and the PSO are very close to that of other methods, indicating that the computational methods are valid for calculating the FOS of a slope. Taking the first slope as an example, VMM calculated the FOS as 1.23, while the EM and the PSO in this study were estimated as 1.23 and 1.24, respectively. The result of employing the EM was that it was more accurate. Compared to the EM, the error from the PSO was also small, indicating that the PSO is feasible for calculating the FOS of a slope.

In these calculations, the step for each searching variable in the EM was 0.1 m; the swarm size of PSO of *N* was 20. The maximum iteration *t*_max⁡_ was 100. Under the same conditions such as the searching range, the total iteration in the EM was much larger than in the PSO. With regard to a large slope, the iteration in EM could reach several tens of millions requiring significant computation time as shown in Table 2.

Therefore, compared with the EM, the PSO had a smaller computation error and it also reduced the computation time. It is a more efficient and accurate method for calculating the FOS of a slope.

The swarm size and iteration steps can significantly affect the computational results in the PSO. To determine the optimum swarm size and iteration steps, this study calculated several swarm sizes, iteration steps and estimated their influence on the convergence speed, computation load, and the results.

**Figure 5:**
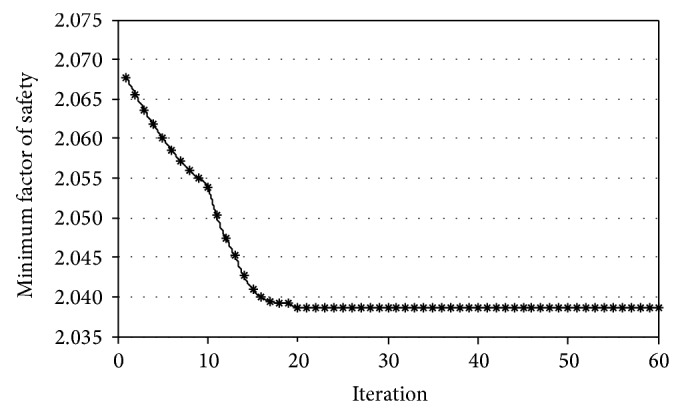
Minimum FOS versus iterations.

**Figure 6:**
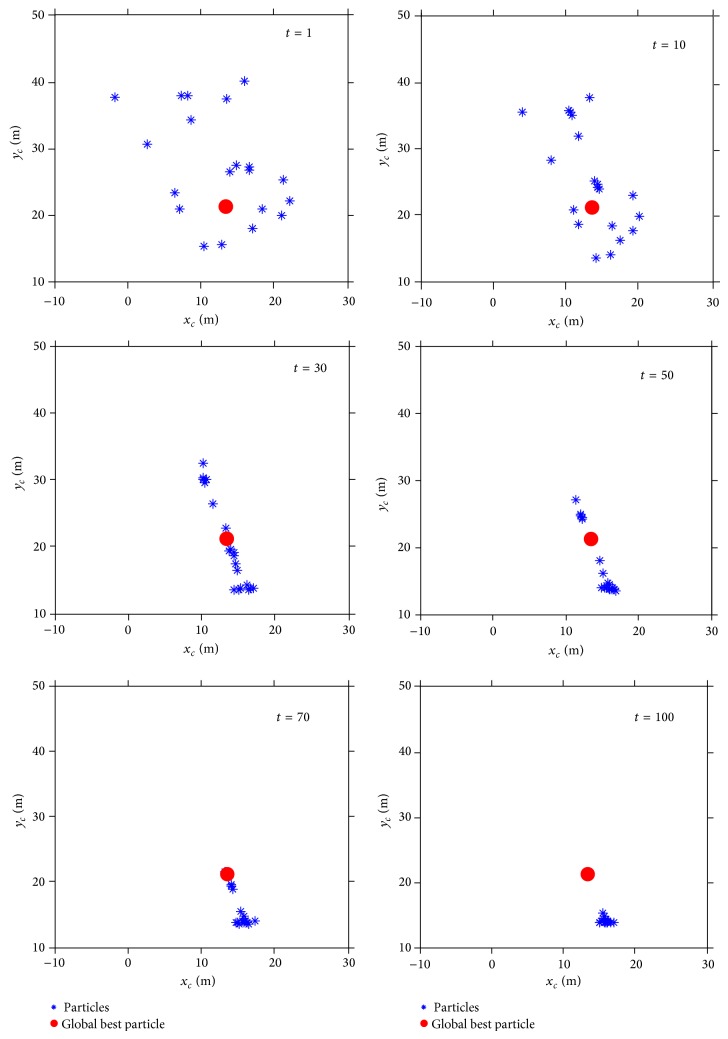
Positions of the particles of different iterations.

Using the second slope as an example, letting *N* = 20, the minimum FOS and location of the particles were investigated as the variation in the iteration steps. The results of this analysis can be seen in Figures 5 and 6. In Figure 5, it appears that the FOS of the slope decreased with the increase of the iteration and reached the minimum value at the 20th iteration. In Figure 6, under the initial condition, that is, *t* = 1, particles dispersed around the global best particle. With the increase of iterations, the particles gradually approached the global best particle. Although the particles were concentrated in the final computation stage, these particles were not clustered in the location of the global best particle, which was primarily due to the particles being caught in the vicinity of the point with the local extreme value in the PSO.

**Figure 7:**
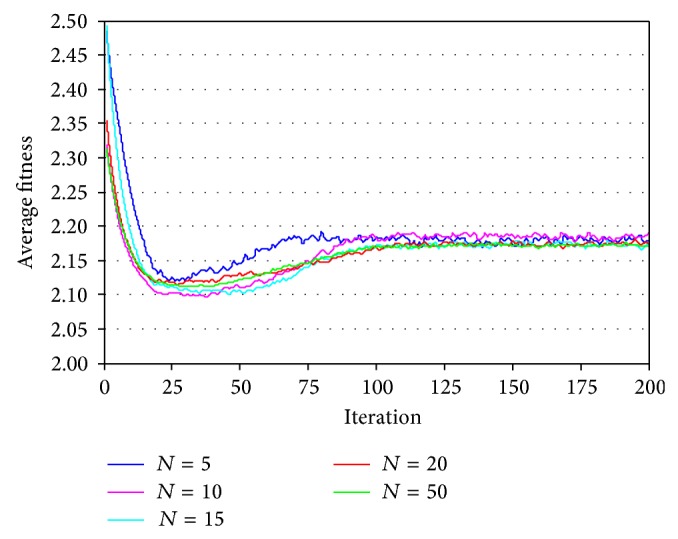
Convergence characteristics of PSO.

Thereafter, letting *t*_max⁡_ = 200, this study analyzed the convergence feature of the PSO with a swarm size *N* of 5, 10, 15, 20, and 50. The results are presented in Figure 7. In the figure, it can be seen that the convergence results were good for the swarm size of 15, 20, and 50. Moreover, for different swarm sizes, the convergence was attained before 100 iterations, indicating that the PSO converged quickly. Therefore, when using the PSO to calculate the slope FOS, selecting *t*_max⁡_ = 100 and *N* = 20 should satisfy the requirements.

### 4.2. Multistage Slope Calculation

**Figure 8:**
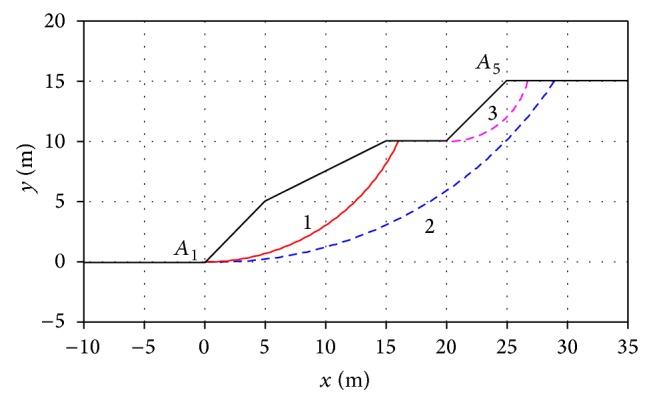
Cross section of slope and computation results.

Figure 8 shows a multistage slope, with the unit weight *γ* = 20 kN/m^3^, cohesion *c* = 10 kPa, and internal friction angle *φ* = 20°. Utilizing the PSO, the minimum FOS was calculated to be 1.076 and the CSS is surface 1 in Figure 8. In addition, two different slip surfaces at completely different locations were calculated with the factors of safety close to the minimum FOS as represented by surfaces 3 in Figure 8. Their factors of safety were 1.118 and 1.156. If reinforcement was performed only to slip surface 1, the slope might slide along surfaces 2 and 3.

**Figure 9:**
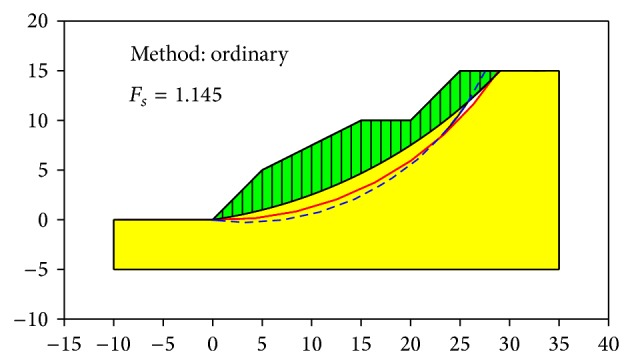
Results of Swedish circle method.

The overall FOS using the Swedish circle method in the software was calculated to be 1.145 as shown in Figure 9. If we used AM2 to calculate the overall FOS of the slope, the slope surface can be assumed to be the line of *A*_1_ *A*_2_. Here, the calculated FOS was 1.009 and the slip surface was the blue dashed line shown in Figure 9. In Figure 9, the red solid line represents the calculated slip surface employing the PSO presented in this study. It can be seen in Figure 9 that the calculated slide-in and slide-out points of the slip surface calculated using the method presented in this paper provided a close result to that obtained using the software. The calculated FOS 1.118 was also close to the calculated value obtained from the software, that is, 1.145, which further validated the accuracy of the method presented in this study.

## 5. Conclusions and Further Research

1. This paper selected the horizontal coordinate of an intercept between the slip surface and the slope top, bottom surface, and the vertical coordinate of the center of the critical arc, as the searching variables. Using the engineering practice to restrain the value range of the searching variables, this study transformed the problem of solving for the minimum FOS of the slope into solving a constrained nonlinear programming problem. The EM and the PSO were easily programmed to solve this problem.
2. The case study of a simple slope indicated that the EM and the PSO provided results that were close to those obtained using the other established methods. In addition, the EM provided the better accuracy of the two. Compared to the EM, the PSO also had small computation errors and significantly reduced the computation load. The PSO can efficiently and accurately calculate the slope FOS.
3. In a case study of a multistage slope, the method presented in this paper estimated the minimum FOS to be 1.076. Two potential slip surfaces were also calculated, with the factors of safety being 1.118 and 1.156, respectively. These proved to be close to the minimum FOS, but the locations of these two surfaces were very different from that of the CSS. Therefore, reinforcement should be carried out for all three surfaces.
4. Through multiple calculations it was found that the PSO tended to converge quickly. Use of the PSO to calculate slope FOS, with *t*_max⁡_ = 100, *N* = 20, usually satisfies the computational requirement. However, with the PSO, the particles could be easily caught at the points with local extreme values, demonstrating that this technique requires further improvement.

## Figures and Tables

**Table 1:** Comparison of safety factor calculated by different methods.

Number	Slope angle (°)	*H* (m)	*C* (kPa)	*φ* (°)	*γ* (kN/m^3^)	Method	FOS	x_c_ (m)	y_c_ (m)
1	39.0	210.0	300.0	25.0	23.0	VMM	1.23	12.7	307.4
						PSO	1.24	12.5	307.2
						EM	1.23	12.9	307.1

2	26.6	13.5	57.5	7.0	17.3	AM1	2.08	—	—
						PSO	2.04	13.3	21.2
						EM	2.04	13.4	21.2

3	18.4	20.0	10.0	20.0	18.0	AM2	1.49	16.4	57.5
						PSO	1.49	16.3	54.7
						EM	1.49	16.4	57.5

4	26.6	12.0	29.0	20.0	19.2	OM	1.93	—	—
						PSO	1.89	8.1	20.1
						EM	1.89	8.1	20.0

5	26.6	25	10	26.6	20	GA	1.33	0	68.8
						PSO	1.31	4.3	58.4
						EM	1.31	4.5	58.0

**Table 2:** Comparison between EM and PSO.

Number	Method	Iteration	Computation time (s)
1	EM	10897394	871.068
	PSO	100	0.423

2	EM	73441	5.292
	PSO	100	0.423

3	EM	4328958	346.002
	PSO	100	0.417

4	EM	892133	70.783
	PSO	100	0.411

5	EM	4651688	376.618
	PSO	100	0.425
